# A Cu(ii) complex supported on Fe_3_O_4_@SiO_2_ as a magnetic heterogeneous catalyst for the reduction of environmental pollutants

**DOI:** 10.1039/d2ra04787j

**Published:** 2022-09-20

**Authors:** Mehdi Khalaj, Maryam Zarandi

**Affiliations:** Department of Chemistry, Islamic Azad University Buinzahra Branch Buinzahra Iran khalaj_mehdi@yahoo.com +98 2834226118 +98 2834226112

## Abstract

Today, the presence of pollutants in the environment has become one of the serious problems and concerns of human beings. To eliminate these pollutants, researchers have made many efforts. One of the most important of these efforts is the reduction of such contaminants in the presence of effective catalysts. Two of the most important and widespread types of these pollutants are nitro compounds and organic dyes. In this paper, we report the synthesis of an efficient and reusable magnetic catalyst using Fe_3_O_4_@SiO_2_ core–shell nanoparticles (NPs), *N*-(4-bromophenyl)-*N*′-benzoylthiourea, and copper(ii). Specifically, the Cu(ii)-*N*-(4-bromophenyl)-*N*′-benzoylthiourea complex supported on Fe_3_O_4_-core magnetic NPs (CM)/SiO_2_-shell (SS) (CM@SS-BBTU-Cu(ii)) has been prepared. CM@SS-BBTU-Cu(ii) was characterized by FT-IR (Fourier transform infrared spectroscopy), XRD (X-ray diffraction), TEM (transmission electron microscopy), HRTEM (high resolution transmission electron microscopy), FFT (fast Fourier transform), VSM (vibrating sample magnetometry), TG-DTA (thermogravimetry-differential thermal analysis), STEM (scanning transmission electron microscopy), EDS (energy-dispersive X-ray spectroscopy), and elemental mapping. The synthesized CM@SS-BBTU-Cu(ii) was applied for the reduction of 4-nitrophenol (4-NP), Congo red (CR), and methylene blue (MB) in the presence of NaBH_4_ (sodium borohydride) at room temperature. CM@SS-BBTU-Cu(ii) can be recycled and reused 5 times. Our results displayed that the performance of the catalyst was not significantly reduced by recycling.

## Abstract 2

Nitro-aromatic-pollutants are hazardous to people and the environment. In this work, the catalytic potential of CM@SS-BBTU-Cu(ii) has been investigated for reduction of nitro group in aqueous media by NaBH_4_.

## Graphical abstract

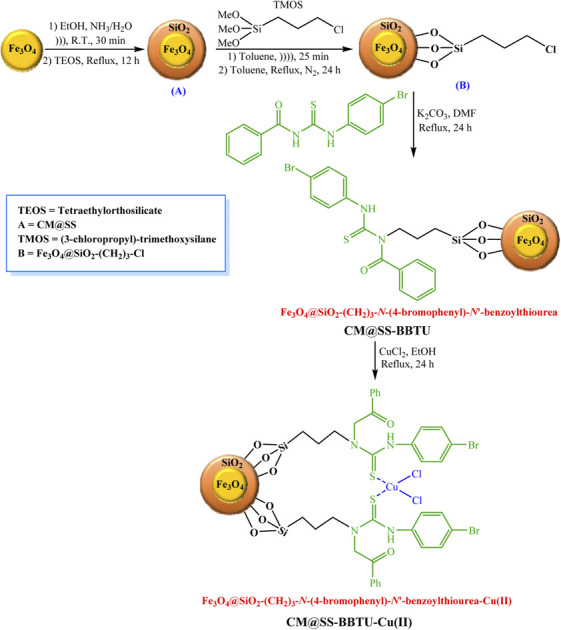

## 1. Introduction

Today, due to the increase in the human population and the corresponding activities, as well as the industrialization of human life, various pollutants have entered the environment.^1–9^ In fact, these pollutants are due to various factors such as agricultural, domestic, industrial activities, *etc.*^10–13^ All over the world, these pollutants, owing to their toxicity, have damaging effects on the environment, humans, and all living organisms.^10–13^ One of the most important types of pollution is water pollution by organic pollutants.^14–16^ As previously indicated, with the industrialization of human life, wastes from various industries pollute water bodies all over the world.^17,18^ These wastes include different industrial wastes, herbicides and pesticide residues, pharmaceutical contaminants, *etc.* The most important pollutants from various industries are organic dyes and nitro compounds. These water pollutants cause serious damage to aquatic animals and, as a result, endanger human health.^19–26^

Nitro compounds are present in wastewater from various industries such as paints, insecticides, pigments, refineries, wood preservatives, *etc.*^27–30^ In accordance with the U.S. Environmental Protection Agency, aromatic nitro compounds are very toxic in nature and damage humans and other living organisms.^31–33^ In addition, organic dyes are very dangerous contaminants owing to their various disadvantages such as highly toxic nature, non-biodegradability and carcinogenicity.^33–36^ As a result, the most important action to take in order to protect the environment and the health of living organisms is for researchers to come up with effective methods to eliminate these pollutants. Of course, the problem is that nitro compounds and organic dyes have high chemical and biological stability and very high water solubility, which makes the conventional methods to eliminate these pollutants ineffective.^33,37^ In recent years, highly toxic and carcinogenic nitroaromatic compounds such as 4-NP have been continuously added to the environment through industrial wastewater. 4-NP is difficult to remove from the environment by commonly used removal methods such as adsorption, photocatalytic degradation, microwave assisted catalytic oxidation, membrane separation, electrocoagulation, and biological treatment due to its stubborn and stable characteristics.^38,39^

One of the most significant routes of chemical industry improvement is the development of novel effective approaches for the synthesis of catalysts.^38–41^ Among other methods to reduce nitro compounds, the catalytic reduction is considered as an appropriate and renewable technique to eliminate nitro compounds and organic dyes because this method has many advantages such as simple work-up, excellent efficiency, cost-effectiveness, and safety.^38,39,42–45^ For the reduction of organic dyes and nitro compounds, it is very necessary to synthesize appropriate and efficient catalysts. Among effective catalysts to eliminate nitro compounds and organic dyes, heterogeneous catalysts are more suitable because they have many advantages such as simple work-up, recyclability, ease of handling, *etc.*^38,39,46,47^ Nowadays, nanotechnology could be used for the synthesis of nanomaterials or nanoparticles, which can be applied in various fields such as food chemistry, biology, drug delivery, energy storage, removal of pollutants, detection of hazardous compounds, catalysis, *etc.*^42–45,48–67^ Heterogeneous catalysts are mainly used in the form of NPs considering their large available catalytic surface.^68–70^ However, metal nanoparticles tend to agglomerate.^33,71–78^ To overcome this problem, many different compounds such as polymers, bentonite, graphene oxide, carbon-based compounds, iron oxide nanoparticles (Fe_3_O_4_ NPs), *etc.* can be applied as effective supports for the preparation of heterogeneous catalysts.^79–83^ Among different metal oxide NPs,^84–89^ Fe_3_O_4_ NPs have engrossed more consideration owing to their many benefits such as great magnetic performance, cost-effectiveness, small size, high specific surface area, and stability.^90,91^ Researchers use silica shells to avoid aggregation of Fe_3_O_4_ NPs. In addition, this delivers many surface Si–OH groups for more modification.^92^ In fact, it has been reported that silica plays an important role as a saving shell to coat Fe_3_O_4_ NPs to synthesize a core–shell (Fe_3_O_4_@SiO_2_) structure.^93^ In recent years, some researchers have synthesized Fe_3_O_4_@SiO_2_-based catalysts for the reduction of organic dyes and nitro compounds.^94–97^

There are several methods for the synthesis of nanostructures^98–111^ and nanocatalysts.^33,38,39,80^ Nowadays, the immobilization of metal complexes on the surface of nanomaterials is considered as an appropriate method to prepare nanocatalysts. In this experimental study, an efficient, novel, and recoverable magnetic catalyst has been synthesized using Cu(ii)-*N*-(4-bromophenyl)-*N*′-benzoylthiourea complex supported on Fe_3_O_4_@SiO_2_ NPs (CM@SS-BBTU-Cu(ii)). In addition, CM@SS-BBTU-Cu(ii) magnetic catalyst has been applied for the reduction of 4-NP, CR, and MB in the presence of NaBH_4_ as the reducing agent at room temperature. According to the experimental results, in the presence of NaBH_4_, CM@SS-BBTU-Cu(ii) catalyst could completely reduce 4-NP (25 mL, 2.5 × 10^−3^ M), CR (25 mL, 1.44 × 10^−5^ M), and MB (25 mL, 3.1 × 10^−5^ M) within 2–90 s. In particular, our results displayed that the performance of CM@SS-BBTU-Cu(ii) catalyst was not significantly reduced after by 5 cycles, demonstrating the favorable stability and durability of CM@SS-BBTU-Cu(ii).

## 2. Experimental

### 2.1. Apparatuses and compounds

All materials were purchased from Merck and Aldrich Chemical Co. and directly utilized as received. Spherically shaped Fe_3_O_4_ NPs with particle sizes of about 20 nm were obtained from Iranian Nanomaterials Pioneers Co. (Mashhad, Iran). The FT-IR spectra were recorded using a PerkinElmer Spectrum 100 FT-IR spectrophotometer. XRD patterns of CM@SS-BBTU and CM@SS-BBTU-Cu(ii) were recorded using a Rigaku Smart Lab system (10–90°). The shape, size, composition, and elemental distribution of CM@SS-BBTU and CM@SS-BBTU-Cu(ii) were determined by TEM and HRTEM (JEM-F200 JEOL), STEM (JEM-F200-TFEG-JEOL Ltd.), and EDS techniques. VSM analysis was performed at 298 K utilizing a SQUID magnetometer 20 (Quantum Design MPMS XL). The thermal studies of CM@SS-BBTU and CM@SS-BBTU-Cu(ii) were performed using a STA 1500 Rheometric-Scientific with a ramping rate of sample 2 °C min^−1^ and flow rate of 120 mL min^−1^. The UV-Vis absorption spectra were recorded on a PerkinElmer LAMBDA35 spectrometer.

### 2.2. Synthesis of *N*-(4-bromophenyl)-*N*′-benzoylthiourea

**Scheme 1:**
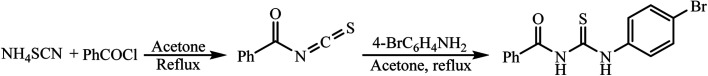
Schematic representation of the synthesis of *N*-(4-bromophenyl)-*N*′-benzoylthiourea.

0.1 mol of ammonium thiocyanate (NH_4_SCN) in acetone was refluxed. Next, 0.1 mol of benzoyl chloride (PhCOCl) in 100 mL of acetone was added to the reaction mixture, which was then stirred for 5 min under reflux conditions. In next step, 0.1 mol of 4-bromoaniline (4-BrC_6_H_4_NH_2_) in 100 mL of acetone was added dropwise to the reaction mixture and refluxing was continued for another 1 h. Afterward, the reaction mixture was slowly poured into water (1500 mL) with vigorous stirring. The obtained product was separated and washed with H_2_O several times and then recrystallized from ethanol (Scheme 1).

### 2.3. Synthesis of Fe_3_O_4_@SiO_2_-*N*-(4-bromophenyl)-*N*′-benzoylthiourea-Cu(ii) (CM@SS-BBTU-Cu(ii))

**Scheme 2:**
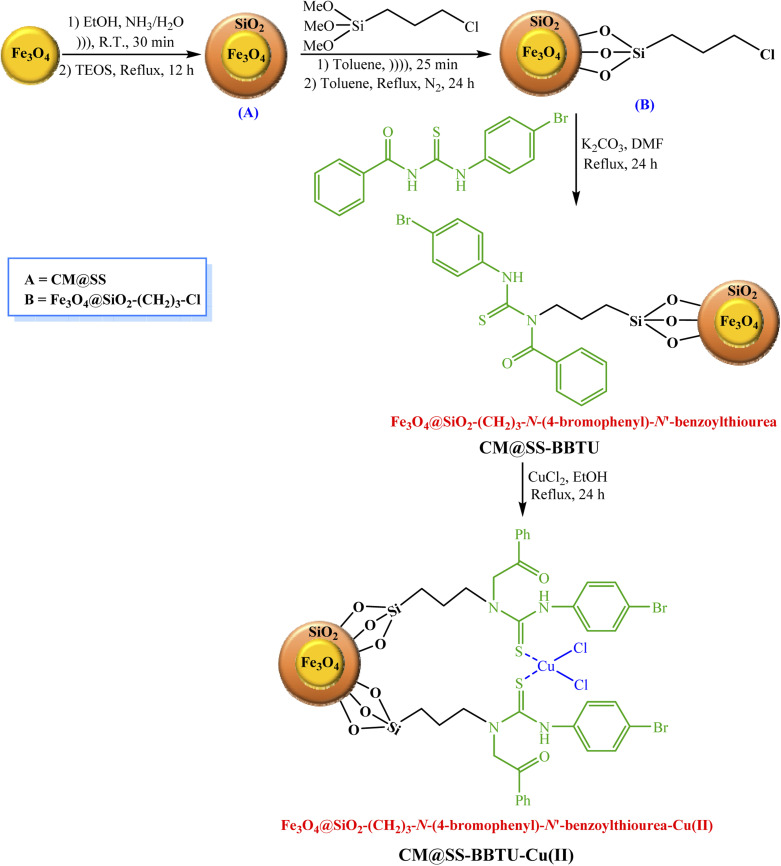
Schematic representation of the synthesis of CM@SS-BBTU-Cu(ii).

According to Scheme 2, 1.0 g of Fe_3_O_4_ NPs was added to a flask (round-bottomed) containing ethanol (80.0 mL, 98%), deionized water (30.0 mL), and 3.0 mL of a solution of 25% ammonia. 1.5 mL of TEOS (tetraethyl orthosilicate) were next added to this solution, which was then refluxed for 12 h for the fabrication of Fe_3_O_4_-core magnetic NPs (CM)/SiO_2_-shell (SS) (A) (CM@SS). Afterward, (A) was uniformly dispersed in 40.0 mL of dry toluene under strong ultrasonication for 25 min. To prepare (B), 3.0 mL of (3-chloropropyl)trimethoxysilane were subsequently added to 1.0 g of (A) and the reaction mixture thus obtained was added to the as-prepared mixture, followed by refluxing under N_2_ atmosphere for 24 h. Fe_3_O_4_@SiO_2_-(CH_2_)_3_-*N*-(4-bromophenyl)-*N*′-benzoylthiourea (CM@SS-BBTU) was then prepared through reacting 2.0 g of chlorofunctionalized NPs with 5.0 mmol of *N*-(4-bromophenyl)-*N*′-benzoylthiourea and 5.0 mmol of K_2_CO_3_ in 50.0 mL of DMF under reflux conditions for 24 h. Finally, 1.0 g of Fe_3_O_4_@SiO_2_-(CH_2_)_3_-*N*-(4-bromophenyl)-*N*′-benzoylthiourea was added to a solution of CuCl_2_·2H_2_O (0.5 g in 50.0 mL of EtOH) and the resulting mixture was refluxed for 24 h (Scheme 2). The synthesized Fe_3_O_4_@SiO_2_-(CH_2_)_3_-*N*-(4-bromophenyl)-*N*′-benzoylthiourea-Cu(ii) nanocatalyst was separated using a magnet, washed with EtOH, dried, and applied as the catalyst in the reduction of 4-NP, CR and MB.

### 2.4. CM@SS-BBTU-Cu(ii)-catalyzed reduction of 4-NP

For reduction test, in a beaker, 25 mL of a solution of 4-NP (2.5 × 10^−3^ M) and 7.0 mg of CM@SS-BBTU-Cu(ii) catalyst were stirred. Next, 25 mL of a freshly prepared solution of NaBH_4_ (0.25 M) was added into the reaction medium and stirring was continued at room temperature. The transformation of 4-NP to the desired 4-aminophenol (4-AP) was monitored by UV-Vis spectroscopy at 317 nm. The disappearance of the yellow color of the reaction medium to colorless indicates that 4-NP has been converted into 4-AP. At the end of the process, CM@SS-BBTU-Cu(ii) nanocatalyst was simply separated by an external magnet from the medium and reactivated by washing with water for recycling studies.

### 2.5. CM@SS-BBTU-Cu(ii)-catalyzed reduction of CR

Furthermore, the catalytic activity of CM@SS-BBTU-Cu(ii) was evaluated for the reduction of CR dye. For this aim, a solution of CR (25 mL, 1.44 × 10^−5^ M) and 7.0 mg of CM@SS-BBTU-Cu(ii) catalyst was stirred in a beaker at room temperature. Afterwards, 25 mL of freshly produced NaBH_4_ (5.3 × 10^−3^ M) was added to the beaker and the resulting mixture was stirred at room temperature. The progress of reduction processes can be followed by UV-Vis spectroscopy at 493 nm. After the solution became colorless and upon completion of the reduction reaction of CR, the nanocatalyst was similarly separated using an external magnet, washed with H_2_O, dried, and then reused in the subsequent runs.

### 2.6. CM@SS-BBTU-Cu(ii)-catalyzed reduction of MB

In a typical experiment, 25 mL of MB solution (3.1 × 10^−5^ M) and 7.0 mg of CM@SS-BBTU-Cu(ii) catalyst were added to a beaker and the resulting mixture was stirred at room temperature. 25 mL of the freshly prepared solution of NaBH_4_ (5.3 × 10^−3^ M) reducing agent were then added to the solution above under stirring at ambient temperature. The reduction of MB was monitored by UV-Vis spectroscopy *via* measuring the changes in the absorbance at the wavelength of 663 nm. It was observed that the blue color of MB solution turned colorless after addition of NaBH_4_ reductant and completion of the catalytic process within 2 s. Upon the completion of reduction reaction, CM@SS-BBTU-Cu(ii) nanocatalyst was separated from the reaction medium using an external magnet, washed with water, dried, and reused in the next cycle.

## 3. Results and discussion

### 3.1. Characterization of CM@SS-BBTU-Cu(ii)

Generally, nanostructures are characterized using various analyses such as XRD, SEM (scanning electron microscopy) TEM, HRTEM and FT-IR.^112–117^ In the present work, the synthesized CM@SS-BBTU-Cu(ii) catalyst was characterized by XRD, FT-IR, TEM, HRTEM, FFT, VSM, TG-DTA, STEM, EDS, and elemental mapping analysis.

**Fig. 1:**
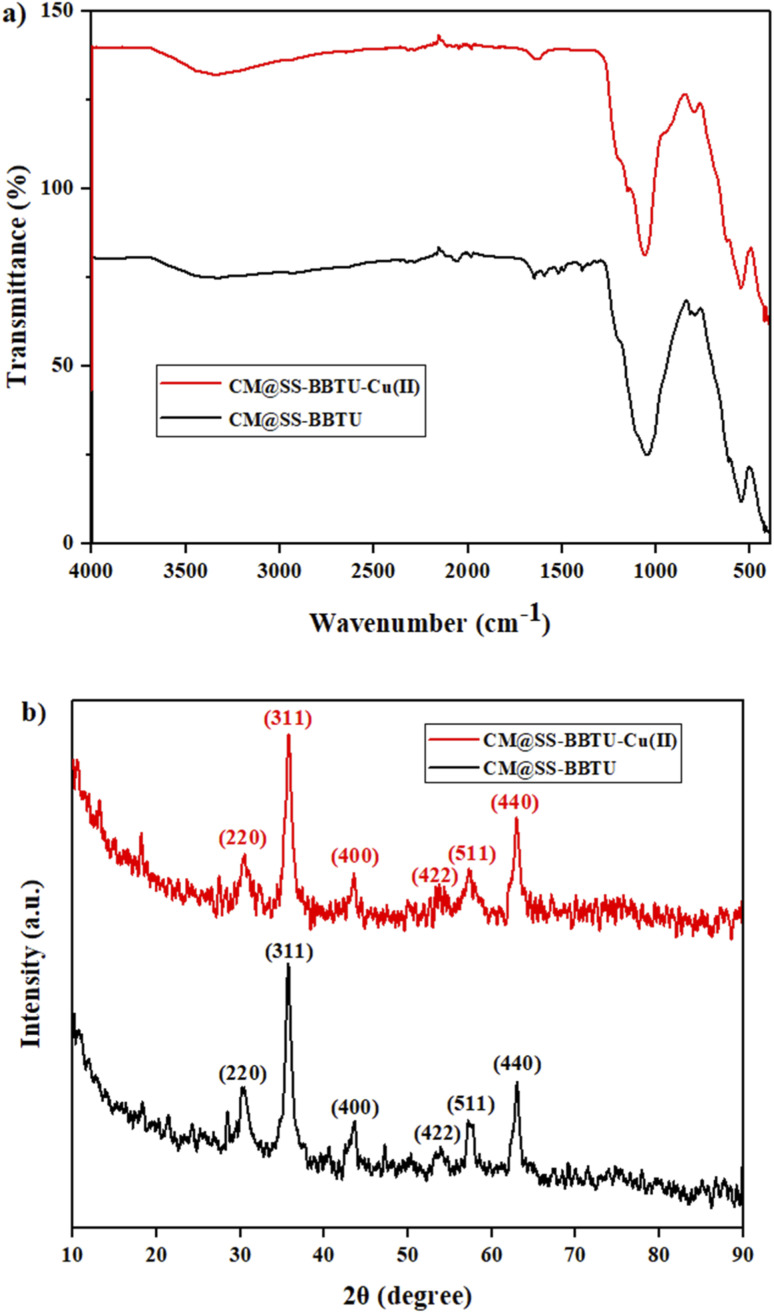
(a) FT-IR spectra and (b) XRD patterns of CM@SS-BBTU and CM@SS-BBTU-Cu(ii).

The functional groups of CM@SS-BBTU and CM@SS-BBTU-Cu(ii) were characterized by FT-IR spectroscopy. Fig. 1a shows the FT-IR spectra of CM@SS-BBTU and CM@SS-BBTU-Cu(ii). The peaks observed at 580, 803, and 1083 cm^−1^ are related to Fe–O, Si–O–Si stretching, and bending vibrations, respectively. The adsorption peak at about 1600 cm^−1^ is associated to C=N bonds in the structure of *N*-(4-bromophenyl)-*N*′-benzoylthiourea. In addition, the broad adsorption peak in the range of 3200–3600 cm^−1^ is ascribed to the –OH and –NH groups. In fact, these peaks confirm the structure of CM@SS-BBTU. The deposition of Cu NPs on the surface of CM@SS-BBTU did not significantly change the functionalities. In other words, after coating copper particles on the surface of CM@SS-BBTU, no new absorption peaks were observed in CM@SS-BBTU-Cu(ii) (Fig. 1a) due to two main reasons including (1) the absorption peak of copper particles is weak because of their low concentration in CM@SS-BBTU-Cu(ii) and (2) the absorption of CM@SS-BBTU overlaps. However, these results indicate that BBTU structure is still stable during the immobilization of Cu NPs.

**Fig. 2:**
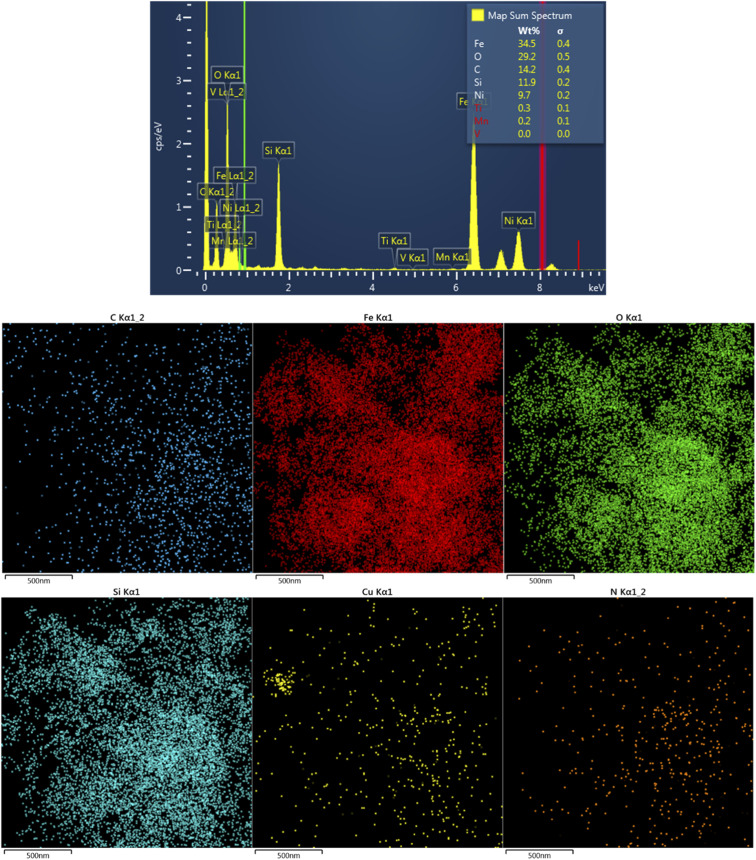
EDS analysis and elemental mapping of CM@SS-BBTU-Cu(ii).

The crystallinity of the synthesized composites was investigated by XRD analysis. This technique can be applied to confirm the presence of Fe_3_O_4_ in the structure of the synthesized catalyst. Fig. 1b shows the XRD patterns of CM@SS-BBTU and CM@SS-BBTU-Cu(ii). The peaks at 2*θ* = 30.1°, 35.4°, 43.1°, 53.5°, 57.9° and 63.0° correspond to the (220), (311), (400), (422), (511), and (440) planes of the cubic Fe_3_O_4_ (JCPDS 19-0629), respectively,^118^ in CM@SS-BBTU structure, confirming the formation of multi-functional magnetic catalyst. The same characteristic peak can also be observed in the XRD pattern of CM@SS-BBTU-Cu(ii) with the same crystallinity, suggesting that the formation of the copper complex has no effect on the properties of CM@SS. According to XRD analysis, no significant peaks corresponding to Cu NPs were observed, which might be ascribed to their low loading. However, the elemental mapping of CM@SS-BBTU-Cu(ii) (Fig. 2) demonstrates the decoration/stabilization of Cu NPs on the surface of CM@SS-BBTU.

CM@SS-BBTU-Cu(ii) catalyst was characterized using EDS and elemental mapping (Fig. 2). However, owing to the good dispersion of copper particles in the structure of CM@SS-BBTU-Cu(ii), the amount of copper cannot be determined by EDS analysis. This is in agreement with XRD analysis. The presence of Cu element on the surface of CM@SS-BBTU was studied by elemental mapping and the results shown in Fig. 2 indicate the distribution of Cu NPs on CM@SS-BBTU surface. Furthermore, the elemental mapping analysis displayed the presence of O, Cu, C, Si, Fe, and N elements in the structure of the catalyst. Accordingly, the presence of Cu NPs verifies the successful synthesis of CM@SS-BBTU-Cu(ii).

**Fig. 3:**
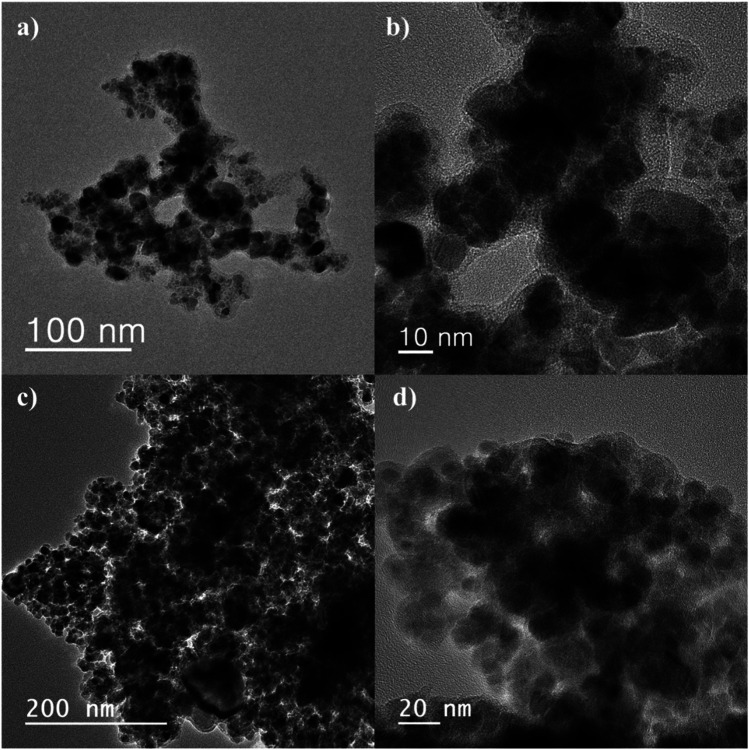
TEM and HRTEM images of CM@SS-BBTU (a, b) and CM@SS-BBTU-Cu(ii) (c, d).

**Fig. 4:**
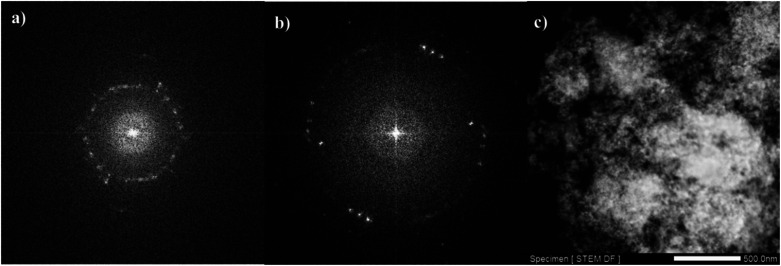
FFT images of CM@SS-BBTU (a) and CM@SS-BBTU-Cu(ii) (b), and STEM image of CM@SS-BBTU-Cu(ii) (c).

TEM analysis is one of the most popular techniques used for detailed characterization of nanostructures through electron microscopy. It reveals size, morphology, dispersion, degree of aggregation, and heterogeneity of nanomaterials. To determine the particle size, TEM and HRTEM analyses were used. Fig. 3a–d show the TEM and HRTEM images of CM@SS-BBTU and CM@SS-BBTU-Cu(ii), respectively. Based on the TEM and HRTEM analyses, CM@SS-BBTU is a good choice for Cu NPs deposition. As displayed in Fig. 3, the Cu(ii) complex is uniformly distributed on CM@SS-BBTU. The average size of Cu(ii) complex is approximately 20 nm. In addition, the FFT images of CM@SS-BBTU and CM@SS-BBTU-Cu(ii) are shown in Fig. 4a and b, respectively, and the STEM images of CM@SS-BBTU-Cu(ii) are shown in Fig. 4c. According to FFT images, Fe_3_O_4_ NPs have crystalline structure. The STEM images display the homogenous structure of CM@SS-BBTU-Cu(ii).

**Fig. 5:**
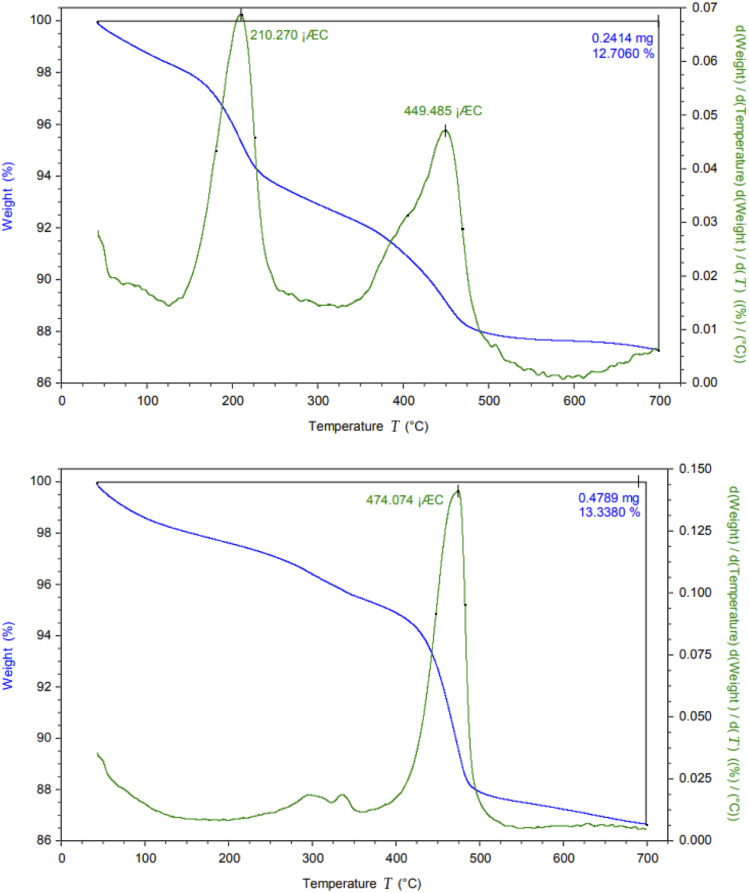
TG/DTA analysis of CM@SS-BBTU (top) and CM@SS-BBTU-Cu(ii) (bottom).

The thermal stability of CM@SS-BBTU and CM@SS-BBTU-Cu(ii) were investigated by TG/DTA at a heating rate of 2 °C min^−1^ from 40 to 700 °C (Fig. 5). In fact, Fig. 5 shows the comparative weight losses of CM@SS-BBTU and CM@SS-BBTU-Cu(ii). In both cases, the weight loss observed below 200 °C is owing to the release of H_2_O or organic solvents in the structures. The loss of weight from 300–500 °C is due to the decomposition of *N*-(4-bromophenyl)-*N*′-benzoylthiourea and other organic groups. Finally, the weight loss above 500 °C is associated with the decomposition of CM@SS-BBTU-Cu(ii) catalyst. As a result, the catalyst possesses high thermal stability in the following catalytic reactions.

**Fig. 6:**
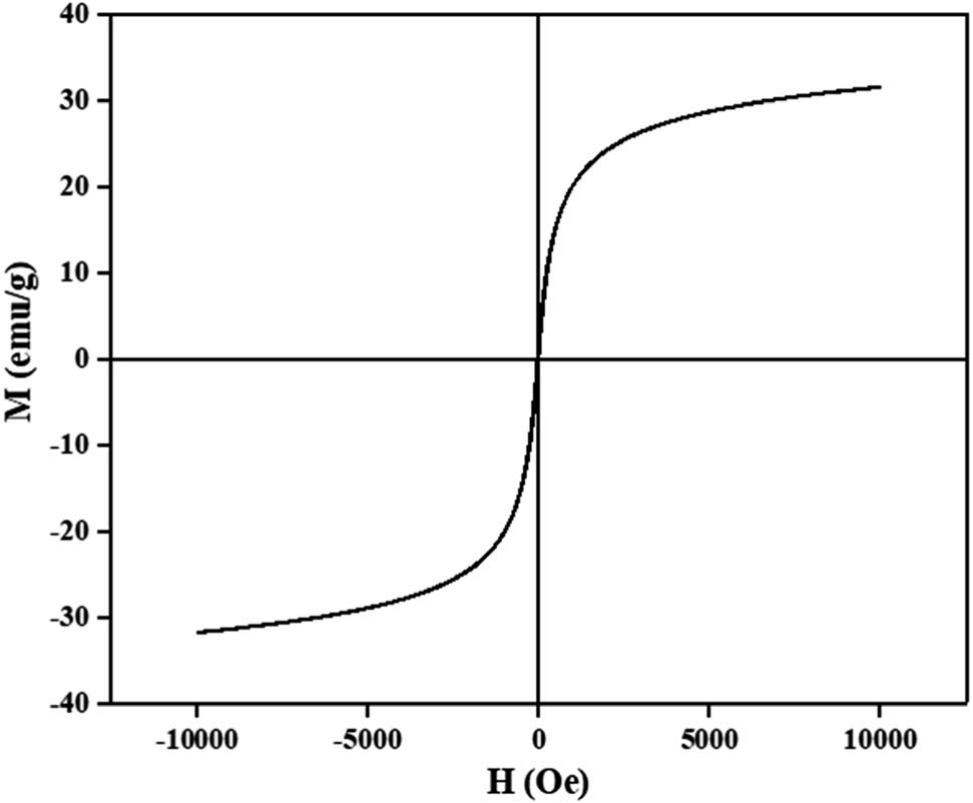
VSM analysis of CM@SS-BBTU-Cu(ii).

The magnetic properties of CM@SS-BBTU-Cu(ii) catalyst were studied using VSM analysis (Fig. 6). The magnetization saturation of CM@SS-BBTU-Cu(ii) catalyst is about 31 emu g^−1^, indicating the superparamagnetic nature of the synthesized catalyst. Although the magnetic saturation is reduced in comparison with Fe_3_O_4_, CM@SS-BBTU-Cu(ii) catalyst could still be simply separated from the electrolysis system using an external magnet. The simple and effective separation of CM@SS-BBTU-Cu(ii) catalyst is necessary for recyclability.

### 3.2. Catalytic activity of CM@SS-BBTU-Cu(ii)

#### 3.2.1. Reduction of 4-NP

To investigate the catalytic activity of CM@SS-BBTU-Cu(ii), the reduction of 4-NP to 4-AP was carried out using NaBH_4_ as a reducing agent at ambient temperature. The experimental results are presented in Table 1. As it is clearly observed, the reduction reaction did not occur in the absence of CM@SS-BBTU-Cu(ii) catalyst (entry 1). Moreover, when CM@SS-BBTU-Cu(ii) was present, but there was no reducing agent (NaBH_4_), the reduction reaction was not complete (entries 2 and 3). In addition, the catalytic activity of CM@SS was investigated in the presence of NaBH_4_, but the reaction was not complete (entry 4). The effects of CM@SS-BBTU-Cu(ii) amount and NaBH_4_ concentration on the catalytic reduction are shown in Table 1. As observed, the reaction time decreased as the amounts of CM@SS-BBTU-Cu(ii) and NaBH_4_ were increased. Here, the amounts of the catalyst and 4-NP concentration were kept constant and the concentration of NaBH_4_ was varied. It was observed that the increase in the concentration of NaBH_4_ increased the reaction rate. The application of 100 mg of NaBH_4_ and 7.0 mg of CM@SS-BBTU-Cu(ii) catalyst resulted in an excellent yield (entry 5).

**Table 1:** 4-NP (2.5 × 10^−3^ M) reduction by CM@SS-BBTU-Cu(ii) and NaBH_4_ at room temperature

Entry	Catalyst (mg)	NaBH_4_ (equivalents)	Time
1	—	100	100 min^a^
2	CM@SS-BBTU-Cu(ii) (5.0)	0.0	240 min^b^
3	CM@SS-BBTU-Cu(ii) (7.0)	0.0	220 min^b^
4	CM@SS MNPs (5.0)	100	30 min^b^
5	CM@SS-BBTU-Cu(ii) (7.0)	100	90 s
6	CM@SS-BBTU-Cu(ii) (5.0)	100	170 s
7	CM@SS-BBTU-Cu(ii) (7.0)	79	155 s
8	CM@SS-BBTU-Cu(ii) (5.0)	79	230 s
9	CM@SS-BBTU-Cu(ii) (7.0)	50	215 s
10	CM@SS-BBTU-Cu(ii) (5.0)	50	255 s
11	CM@SS-BBTU-Cu(ii) (3.0)	100	5 min

a No reaction.

b Not completed.

**Fig. 7:**
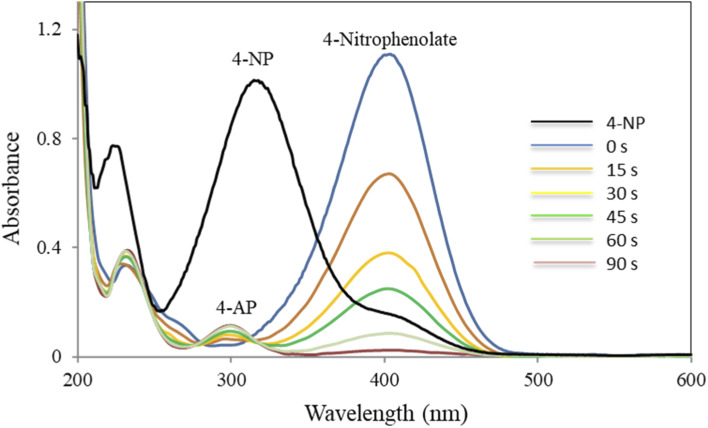
UV-Vis spectra of the reduction of 4-NP in the presence of NaBH_4_ and CM@SS-BBTU-Cu(ii).

The progress of the reduction process was followed by UV-Vis spectroscopy (Fig. 7). As shown in Fig. 7, 4-NP solution displayed a specific SPR band at 317 nm. A visual color change of the light-yellow solution of 4-NP to bright yellow 4-nitrophenolate ion (4-NPT) was observed in the presence of NaBH_4_ along with a redshift of the absorption peak from 317 (broad) to 400 nm. It is important to note that removing H_2_ by addition of NaBH_4_ to 4-NP solution resulted in 4-NPT formation. A shown in Table 1 (entry 1), no further reaction occurred in the absence of CM@SS-BBTU-Cu(ii), which shows NaBH_4_ alone is not sufficient for the reduction of 4-NP. In the absence of CM@SS-BBTU-Cu(ii) nanocatalyst, the reduction of 4-NP to 4-AP did not occur even after 100 min. and the absorption peak of 4-NPT at 400 nm remained unchanged for a long time without change in intensity. When CM@SS-BBTU-Cu(ii) catalyst was added into the reaction mixture, the peak at 400 nm gradually decreased and a typical absorption peak appeared at 297 nm due to 4-AP formation.

**Scheme 3:**
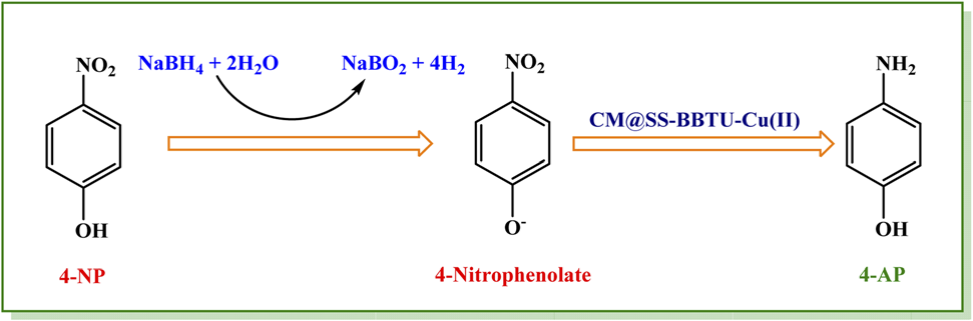
The mechanism for catalytic reduction of 4-NP in the presence of NaBH_4_.

Scheme 3 shows the reaction mechanism for the reduction of 4-NP. Inspired from previous reports,^21,23,24,28^ the reduction of 4-NP to 4-AP using excess NaBH_4_ in the aqueous medium involves electron transfer (ET) from an electron donor to an electron acceptor. As it is clearly observed, CM@SS-BBTU-Cu(ii) has a noteworthy role in the electron transfer effect between the nitro and BH_4_^−^ groups as the electron acceptor and donor, respectively. Here, the Cu NPs decorated onto CM@SS-BBTU decrease the kinetic barrier for 4-NP reduction by acting as an electron relay between BH_4_^−^ donor and nitro acceptor, which are adsorbed on the surface of the catalyst. As shown in Scheme 3, in the first step, the adsorption of BH_4_^−^ ion and 4-NP occurs on the catalytic active sites available on the surface of CM@SS-BBTU-Cu(ii) catalyst *via* π–π stacking interactions. In the next stage, the transfer of electron from BH_4_^−^ and 4-nitrophenolate ions happens. Finally, the desorption of 4-AP from the surface of CM@SS-BBTU-Cu(ii) occurs to free up the active sites and the reduction reaction cycle starts over.

#### 3.2.2. Reduction of CR and MB

**Scheme 4:**
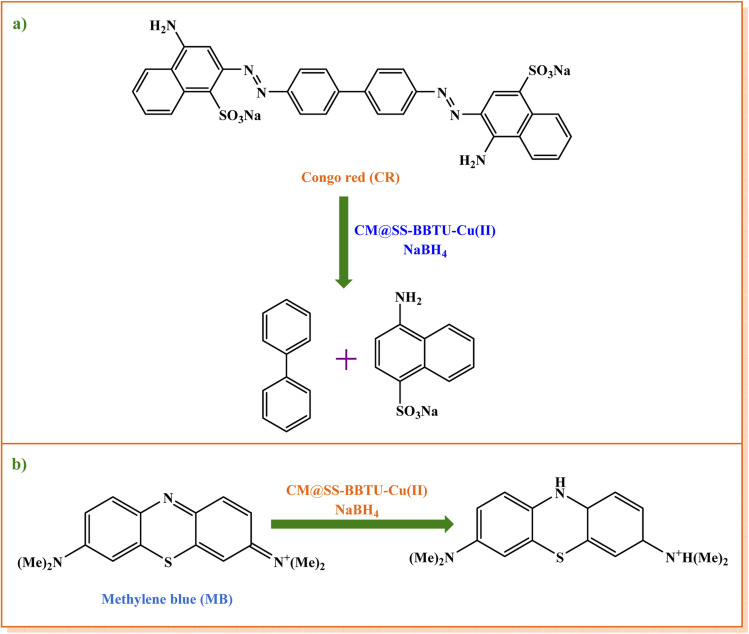
CM@SS-BBTU-Cu(ii)-catalyzed reduction of (a) CR and (b) MB using NaBH_4_.

**Fig. 8:**
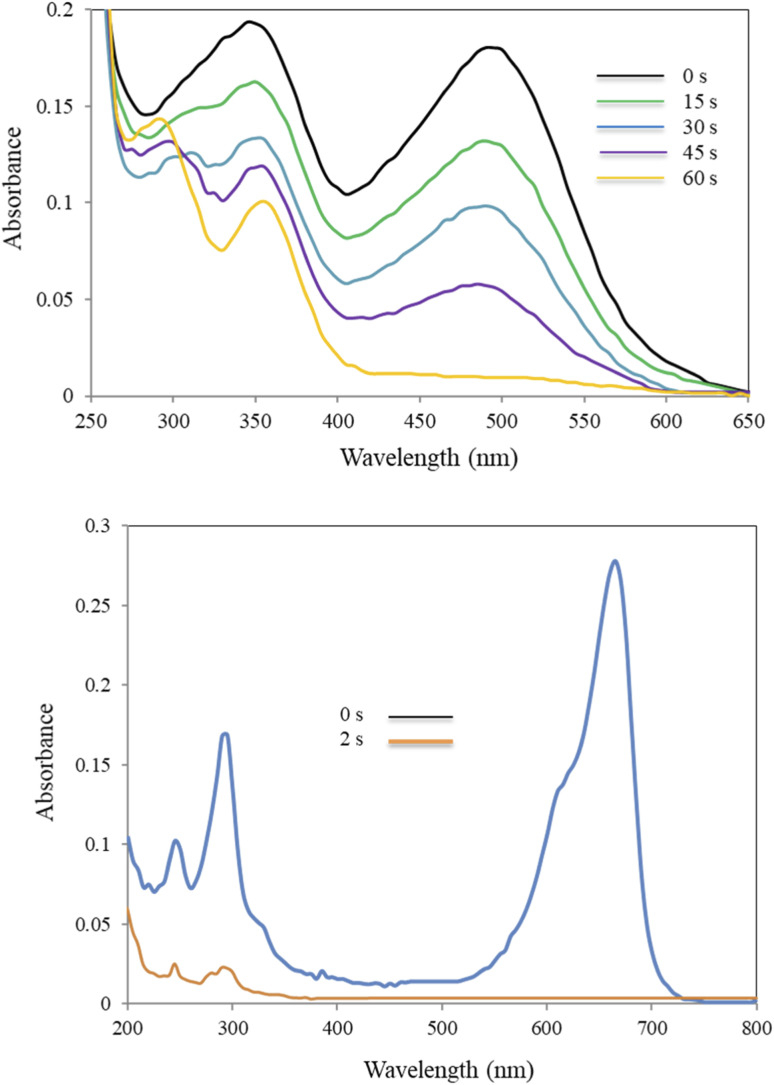
Alterations in UV-Vis spectra of CR (top) and MB (bottom) with time in NaBH_4_-mediated catalytic reduction.

Moreover, the catalytic activity of CM@SS-BBTU-Cu(ii) was investigated for the reduction of CR and MB using NaBH_4_ in the aqueous medium at room temperature (Scheme 4). Table 2 shows the experimental results. According to the results shown in Table 2, the effect of the catalyst on CR and MB reduction indicated that the process did not proceed even after 120 min in the absence of CM@SS-BBTU-Cu(ii) (entries 1 and 7). In addition, the reduction reaction was not complete in the absence of NaBH_4_ as the reducing agent (entries 2 and 6). These observations indicated that the presence of both CM@SS-BBTU-Cu(ii) catalyst and NaBH_4_ was necessary to perform the reduction. The optimal result was attained using 7.0 mg of CM@SS-BBTU-Cu(ii) catalyst, and reaction times of 2 and 60 s for MB and CR, respectively (entries 5 and 10). The progress of the reduction reaction of CR and MB was monitored using UV-Vis spectroscopy (Fig. 8). According to the UV-Vis spectra of CR and MB, the SPR bands at 493 and 663 nm disappeared after 60 and 2 s for CR and MB, respectively.

**Table 2:** Reduction reaction of CR and MB using CM@SS-BBTU-Cu(ii) and NaBH_4_ at room temperature

Entry	CM@SS-BBTU-Cu(ii) (mg)	Dye (M)	NaBH_4_ (M)	Time
1	0.0	MB (3.1 × 10^−5^)	5.3 × 10^−3^	120 min^a^
2	7.0	MB (3.1 × 10^−5^)	0.0	60 min^b^
3	3.0	MB (3.1 × 10^−5^)	5.3 × 10^−3^	10 s
4	5.0	MB (3.1 × 10^−5^)	5.3 × 10^−3^	6 s
5	7.0	MB (3.1 × 10^−5^)	5.3 × 10^−3^	2 s
6	7.0	CR (1.44 × 10^−5^)	0.0	70 min^b^
7	0.0	CR (1.44 × 10^−5^)	5.3 × 10^−3^	120 min^a^
8	3.0	CR (1.44 × 10^−5^)	5.3 × 10	105 s
9	5.0	CR (1.44 × 10^−5^)	5.3 × 10	76 s
10	7.0	CR (1.44 × 10^−5^)	5.3 × 10^−3^	60 s

a No reaction.

b Not completed.

Moreover, the catalytic activity of the synthesized CM@SS-BBTU-Cu(ii) catalyst was compared with those of some heterogeneous catalysts^33,119–130^ recently used in the reduction of MB in order to show the advantage of our catalyst system (Table 3). The results showed that CM@SS-BBTU-Cu(ii) was better than previously reported catalysts in terms of reaction time. It is believed that the catalyst has tremendous activity for the reduction of MB, which may be due to CM@SS-BBTU as an ideal platform for loading Cu NPs, avoiding aggregation and providing uniform dispersion.

**Table 3:** Comparative study of the efficiency of our catalyst with those of previously reported catalytic systems in the NaBH_4_-mediated reduction of MB

Entry	Catalyst	Reaction conditions	Reaction time	Ref.
1	Au NPs/ZSM-5 (1 mg)	MB (2.5 mL, 25 ppm), NaBH_4_ (200 μL, 0.2 M)	4 min	119
2	Fe_3_O_4_-Ag (1 mg)	MB (2 mL, 40 mg L^−1^), NaBH_4_ (0.1 mL, 0.1 M)	18 min	120
3	Montmorillonite/g-C_3_N_4_/Au NPs (250 mg L^−1^)	MB (100 mL, 25 mg L^−1^), NaBH_4_ (0.05 M), pH 11, 35 °C	30 s	121
4	SiNWAs-Cu (1 × 1 cm^2^)	MB (25 mL, 5 × 10^−5^ M), NaBH_4_ (0.01 g)	10 min	122
5	Au-PANI nanocomposite (40 μL, 1 mg mL^−1^)	MB (1 mL, 1 mM), NaBH_4_ (10 mM)	8 min	123
6	GO/Fe_3_O_4_@Dop/AuNPs (1 mg)	MB (2 mL, 3.12 × 10^−5^ M), NaBH_4_ (2 mL, 0.2 M)	20 s	124
7	Montmorillonite@GO@Au (100 mg L^−1^)	MB (100 mL, 25 mg L^−1^), NaBH_4_ (0.05 M)	6 min	125
8	Pd/CS/γMnO_2_ (8 mg)	MB (1 mL, 1.3 × 10^−5^ M), NaBH_4_ (0.1 mL, 0.06 M)	17 s	33
9	Fe_3_O_4_@PANI-Au (1 mg)	MB (5 mL, 3.1 × 10^−5^ M), NaBH_4_ (2 mL, 5.3 × 10^−3^ M)	60 s	126
10	Pd-CS-g-C_3_N_4_ (5 mg)	MB (1 mL, 1 × 10^−5^ M), NaBH_4_ (0.1 mL, 0.05 M)	5 s	127
11	Magnetic polydopamine-Cu (0.4 mg)	MB (0.1 mL, 200 ppm), NaBH_4_ (0.66 mL, 3 M)	5 min	128
12	Cu(NPs)/β-chitin/dicalcium phosphate anhydrous (10 mg)	MB (2 mL, 16 ppm), NaBH_4_ (0.5 mL, 0.04 M)	4 min	129
13	GA-Sch-Pd (5 mg)	MB (1 × 10^−5^ M), NaBH_4_ (1 mL, 0.05 M)	5 s	130
14	CM@SS-BBTU-Cu(ii) (7 mg)	MB (25 mL, 3.1 × 10^−5^ M), NaBH_4_ (25 mL, 5.3 × 10^−3^ M)	2 s	This work

## 4. Recyclability of CM@SS-BBTU-Cu(ii)

**Fig. 9:**
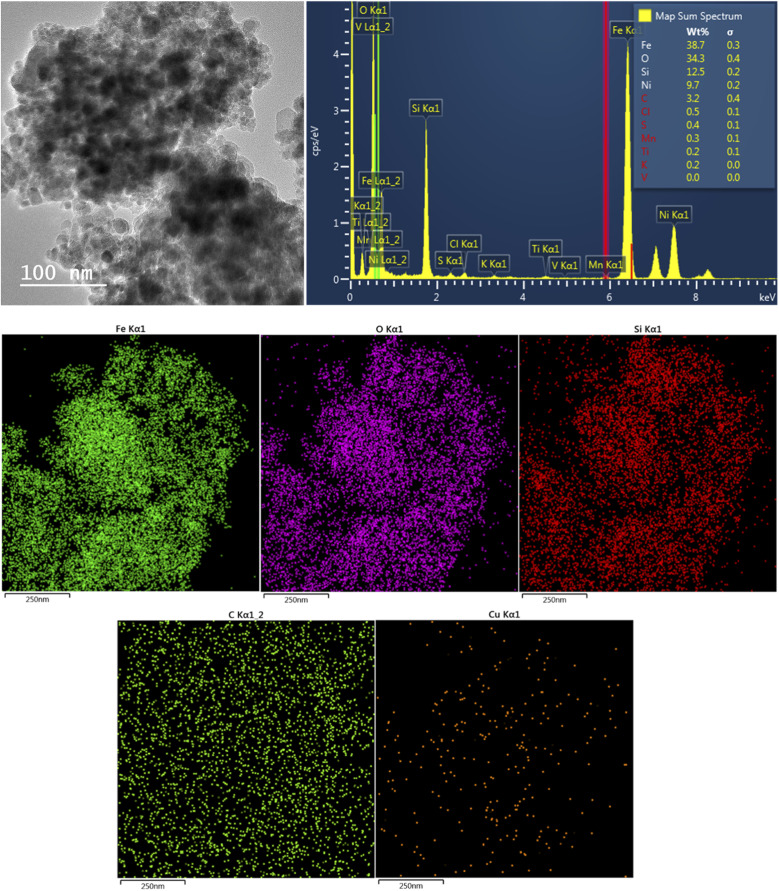
TEM, EDS (top) and elemental mapping (bottom) analysis of the reused CM@SS-BBTU-Cu(ii) catalyst.

The capability of the separation of the catalyst from the mixture of reaction is one of the most noteworthy properties of heterogeneous catalysts. Thus, magnetic catalysts are very useful owing to their simple separation. The synthesized CM@SS-BBTU-Cu(ii) can be easily filtered from the reaction mixture using an external magnet. For this aim, the reduction of 4-NP was selected as the model reaction. Following each recycling test, a new batch of 4-NP solution, styrene, NaBH_4_, and the recovered catalyst were added to a beaker, and the reaction mixture was stirred under optimal conditions. After completion of the reduction, CM@SS-BBTU-Cu(ii) catalyst was filtered, washed with water several times, dried at 70 °C for 4 h, and reused in the following cycle under identical reaction conditions. After each cycle, the activity of CM@SS-BBTU-Cu(ii) catalyst was measured by UV-Vis analysis. Our results show that CM@SS-BBTU-Cu(ii) can be recycled 5 times with high performance and efficiency without noteworthy reduction in the catalytic activity. According to the UV-Vis spectra, the time required for the reduction of 4-NP using NaBH_4_ was found to be almost the same even on the fifth reaction sequence (100% conversion, 95 s). This result showed that Cu NPs are not detached from CM@SS-BBTU surface during the reaction, which indicates that CM@SS-BBTU-Cu(ii) is a very stable catalyst. For more confirmation, to check the heterogeneity of catalyst, which is an important factor, the filtrate in each cycle was analyzed by inductively coupled plasma atomic emission spectroscopy (ICP-AES). It was observed that less than 0.1% of the total amount of Cu was lost into solution during the course of the reduction. In addition, in order to justify the stability of CM@SS-BBTU-Cu(ii) catalyst, the morphological/structural characteristics of the used catalyst were compared to those of the fresh one by performing elemental mapping, EDS, and TEM analyses following the reusability tests. Fig. 9 shows the TEM, EDS, and elemental mapping images of the reused CM@SS-BBTU-Cu(ii) catalyst. As exhibited in Fig. 9, no evident alteration in the chemical composition, structure, morphology, and size of CM@SS-BBTU-Cu(ii) were observed.

## 5. Conclusion

In this experimental work, an efficient and novel Cu(ii)-*N*-(4-bromophenyl)-*N*′-benzoylthiourea complex supported on Fe_3_O_4_@SiO_2_ NPs (CM@SS-BBTU-Cu(ii)) has been synthesized. Fe_3_O_4_@SiO_2_ NPs acts as important magnetic support for the immobilization of Cu(ii) complex. The prepared CM@SS-BBTU-Cu(ii) was analyzed by XRD, FT-IR, TEM, HRTEM, FFT, VSM, TG-DTA, STEM, EDS, and elemental mapping analyses. The catalytic properties of CM@SS-BBTU-Cu(ii) for the reduction of 4-NP, CR, and MB by NaBH_4_ reducing agent at ambient temperature were examined. The best results were achieved when 7.0 mg of CM@SS-BBTU-Cu(ii) we used and the reduction reaction times were 90, 60, and 2 s for 4-NP, CR, and MB, respectively. CM@SS-BBTU-Cu(ii) catalyst can be recycled 5 times without remarkable decrease in the catalytic performance.

## Conflicts of interest

The authors declare that they have no declaration of interest to influence the work reported in this paper.

## Supplementary Material
